# Field-Controlled Microrobots Fabricated by Photopolymerization

**DOI:** 10.34133/cbsystems.0009

**Published:** 2023-06-06

**Authors:** Xiyue Liang, Zhuo Chen, Yan Deng, Dan Liu, Xiaoming Liu, Qiang Huang, Tatsuo Arai

**Affiliations:** ^1^ School of Mechatronical Engineering, Beijing Institute of Technology, Beijing 100081, China.; ^2^ Center for Neuroscience and Biomedical Engineering, The University of Electro-Communications, Tokyo 182-8585, Japan.

## Abstract

Field-controlled microrobots have attracted extensive research in the biological and medical fields due to the prominent characteristics including high flexibility, small size, strong controllability, remote manipulation, and minimal damage to living organisms. However, the fabrication of these field-controlled microrobots with complex and high-precision 2- or 3-dimensional structures remains challenging. The photopolymerization technology is often chosen to fabricate field-controlled microrobots due to its fast-printing velocity, high accuracy, and high surface quality. This review categorizes the photopolymerization technologies utilized in the fabrication of field-controlled microrobots into stereolithography, digital light processing, and 2-photon polymerization. Furthermore, the photopolymerized microrobots actuated by different field forces and their functions are introduced. Finally, we conclude the future development and potential applications of photopolymerization for the fabrication of field-controlled microrobots.

## Introduction

Microrobots have become one of the most promising tools in the biomedical field due to their increasing abilities of minimally invasive surgery, targeted therapy, and cell manipulation [1–4]. Besides, microrobots also show great potential in environmental fields, including decontamination and toxicity screening under conditions too dangerous or too small for humans to access [5,6]. In particular, the untethered microrobots controlled by multiple physical fields, such as magnetic [7], optical [8], acoustic [9], and electric fields [10], show better performance and a more comprehensive range of applications. With the increasing application fields and task requirements, field-controlled microrobots are becoming more intelligent and talented, which mainly relies on the complex purposeful 2-dimensional (2D) and 3D structure design of the microrobots. Thus, the microfabrication technology for fabricating the robots on a microscale with precise 2D/3D arbitrary structures is the key issue in the development and promotion of the field-controlled microrobots.

The untethered field-controlled microrobots can be used in biological surgery and medical diagnosis/treatment due to its low damage and invasiveness to targets. To date, various fabrication methods have been used to produce field-controlled microrobots. Researchers used a membrane template-assisted electrodeposition method to fabricate physically controlled microrobots [11–14]. The physical vapor deposition technology is also used to fabricate field-controlled microrobots [15,16]. Although these methods enumerated can be used to fabricate field-controlled microrobots, however, the fabricated microrobot shapes are limited to simple structures such as spherical and cylindrical shapes.

In recent years, multiple 3D printing technologies have been used to fabricate field-controlled microrobots, for example, the microrobots with complex structural magnetic field control using direct ink printing technology [17,18] and fused deposition modeling technology [19]. These 3D printing technologies show great advantages in fabricating field-controlled microrobots with complex structures. However, these printing techniques are insufficient in the precision required by the microrobots to operate single cells and deliver the drug in microvessels.

Photopolymerization is a widely used 3D printing technology. Typically, it fabricates the structures through selectively polymerizing liquid photopolymer by light following layer-by-layer. The photopolymerization technology outperforms the other fabrication methods in terms of printing velocity, accuracy, and surface quality [20–22]. With the increasing requirements on multiple functions and access to the microenvironment, the structure of field-controlled microrobots is becoming smaller and more complex. Employing photopolymerization in the fabrication of field-controlled microrobots provides an ideal solution. Although researchers have made many efforts to fabricate field-controlled microrobots by photopolymerization [23,24], few articles introduce this field.

**Fig. 1.**
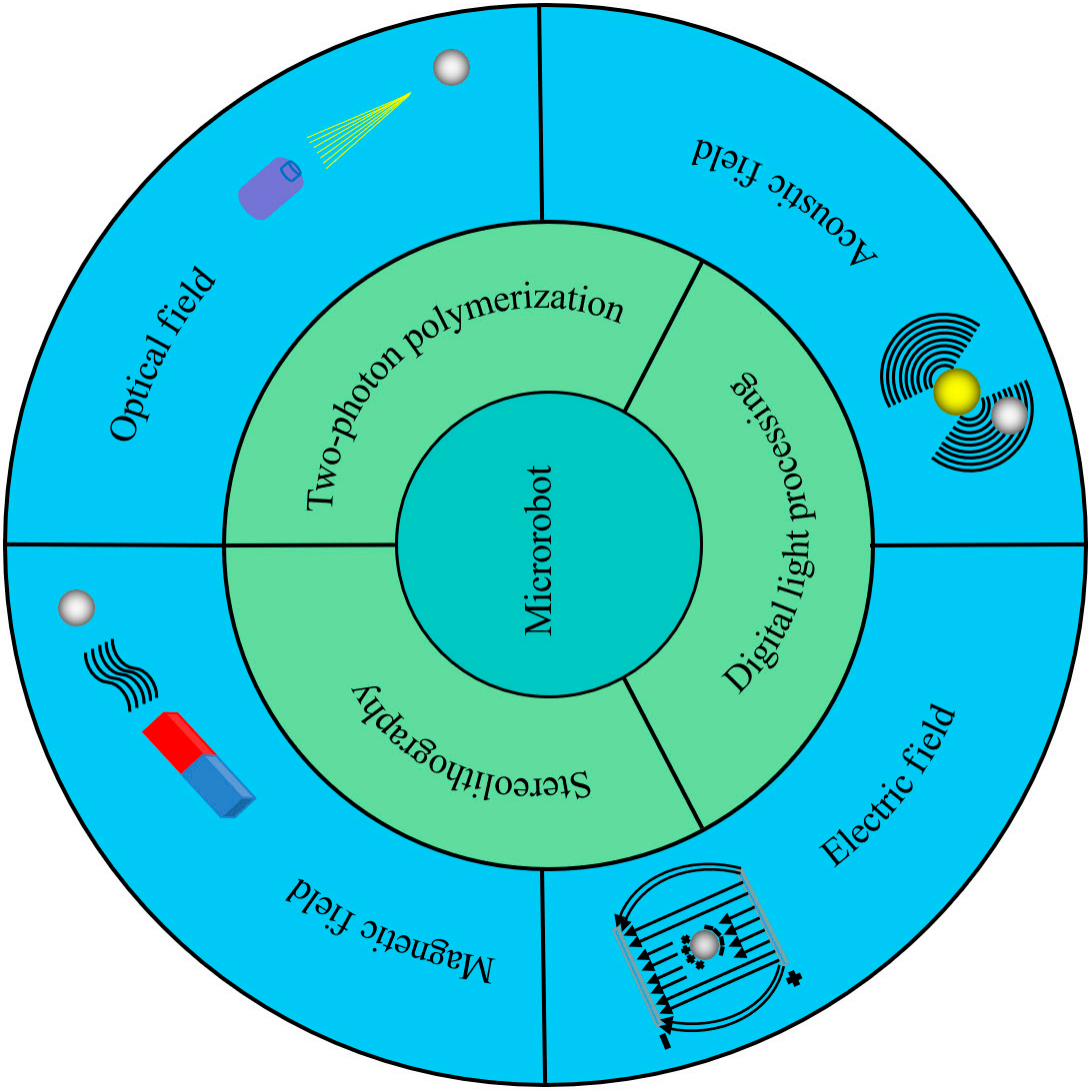
Schematic illustration of field-controlled microrobots fabricated by photopolymerization.

In this review, we summarize the recent research on field-controlled microrobots fabricated by photopolymerizationas shown in Fig. 1. First, the typical photopolymerization technologies for microrobot fabrication are introduced in the Fabrication Based on Photopolymerization section. Then, the Photopolymerized Microrobots Controlled by Varied Field Forces section analyses the recent progress of photopolymerized microrobots controlled by magnetic fields, optical, acoustic, and electric fields. Finally, we summarize the fabrication of field-controlled microrobots based on photopolymerization and look forward to the future development of photopolymerization for microrobot fabrication in the Conclusion and Outlook section. We believe that photopolymerization technology promotes the progress of field-controlled microrobots in many terms, including manipulation accuracy, function, flexibility, and size. Meanwhile, the requirements on the structure, material, and size of field-controlled microrobots also positively accelerate the development of photopolymerization technology.

## Fabrication Based on Photopolymerization

3D printing is a process that fabricates 3D objects layer-by-layer. Photopolymerization, as one of the widely used printing technologies, utilizes the light source to irradiate a reservoir filled with a photosensitive liquid polymer, which cures layer-by-layer at a specific location to form a 3D solid structure [25]. Photopolymerization has the advantages of fast-printing velocity, high accuracy, and high surface quality that can be used to fabricate high-quality components with smooth surfaces and fine details [26]. Typical photopolymerization technologies such as stereolithography (SLA), digital light processing (DLP), and two-photon polymerization (TPP) are described in this section.

### Stereolithography

**Fig. 2.**
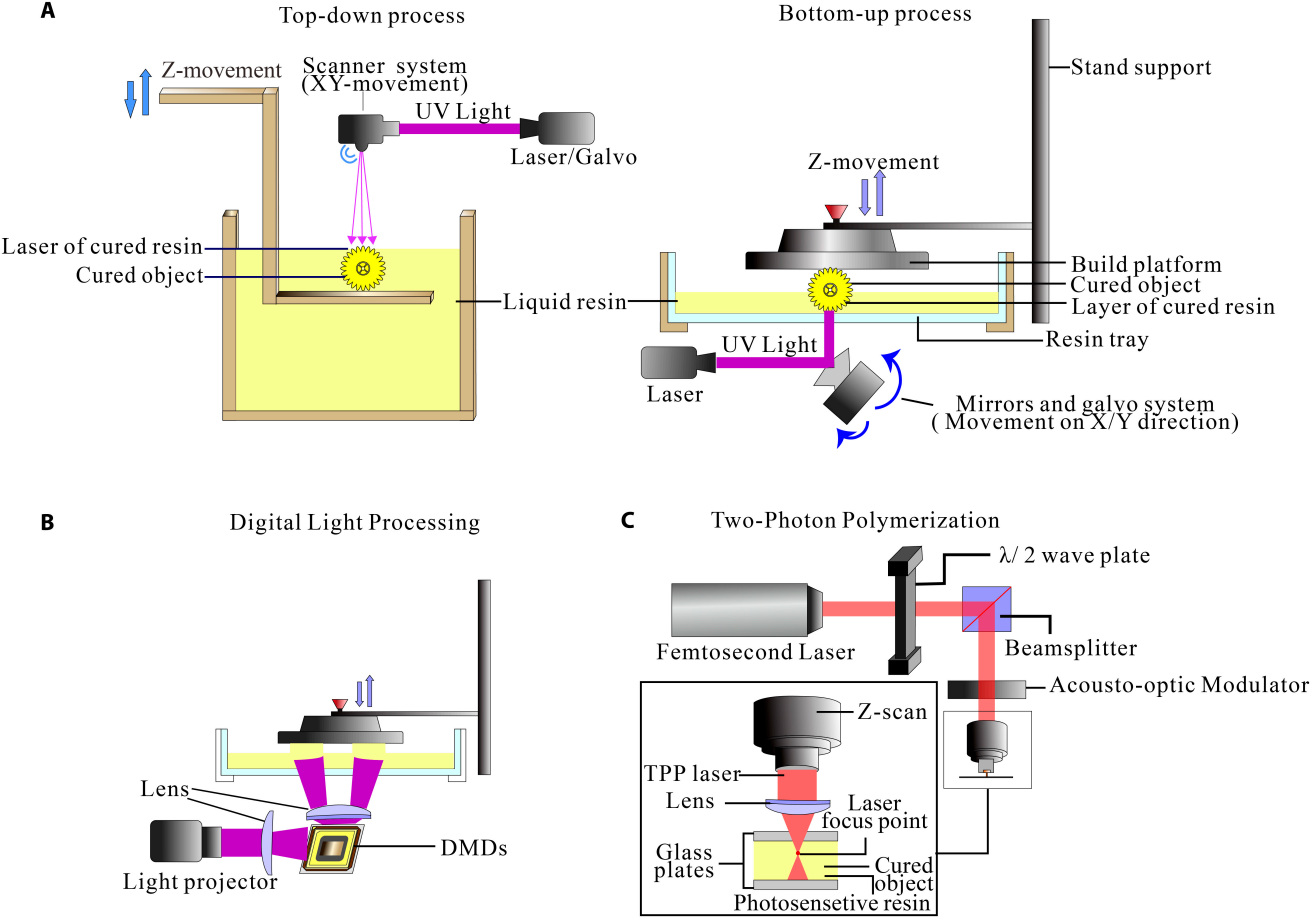
Fabrication technology by photopolymerization. (A) Schematic of SLA. (B) Schematic of DLP. (C) Schematic of TPP.

SLA is the first 3D printing technology created on the basis of Chuck Hull’s view [27]. SLA technology first appeared as a top-down printing method [28], and then the bottom-up printing method is designed to address the size limitations of top-down machines (Fig. 2A). SLA is a method that uses a mobile photon source to activate the photopolymerization of photocurable resin and successively prints solid layer-by-layer [27]. The printed 3D structure is formed by point-by-point illumination and guided by 3D interpretation software [29,30]. SLA is a relatively slow production technology due to the refilling of the materials when printing layer-by-layer, as well as material viscoelastic limitations. However, benefit from the single-point irradiation that controls the laser, SLA can precisely produce a wide variety of objects. Currently, the resolution of SLA printing can be as satisfactory as 10 μm, and it is possible to fabricate microrobots with SLA printing.

### Digital light processing

DLP, as a particular type of SLA technology [31–33], adopts a digital micromirror device (DMD) chip as the core component (Fig. 2B). DMD can selectively reflect light to print the targets layer-by-layer [34–37]. The printing accuracy of DLP depends mainly on the quality of DMD chips. Compared with the traditional SLA technique, DLP improves the velocity of sample fabrication [38]. Moreover, DLP technology has more selectivity for light sources, i.e., light-emitting diode lamps, mercury lamps, and lasers. However, the resin materials used in DLP printing are expensive, and the stiffness and heat resistance after molding are poor. The print resolution of DLP is heavily limited by the number and size of micromirrors in the DMD.

### Two-photon polymerization

TPP technology, also known as direct laser writing (DLW), has become increasingly popular in 3D printing due to its high precision peculiarity [39–41]. TPP uses a femtosecond near-infrared pulse laser as the light source that induces the polymerization of the photosensitive material by absorbing the energy of 2 photons inside the material (Fig. 2C) [42]. Moreover, the TPP technology can break the optical diffraction limit and has a spatial resolution of nanometer level [43,44]. What is unique about TPP compared with other photopolymer printing technologies is that the overall fabrication is designed in the way of hole-in-hole instead of layer-by-layer aggregation [39,45]. However, the kinds of photosensitive materials used for TPP technology are limited, and the processing of micro- and nanoscale takes a long time, so it is difficult to use it to process large-scale products.

## Photopolymerized Microrobots Controlled by Varied Field Forces

Various field forces have been employed to drive the microrobots fabricated by polymerization. In this section, recent advances in actuating microrobots via magnetic, optical, acoustic, and electric fields and the respectively utilized photopolymerization methods are discussed in detail.

### Magnetically controlled microrobots

Magnetic field actuation is promising for microrobots locomotion due to its noninvasive control and navigation method [1,46]. A magnetically controlled microrobot system consists of microrobots made of magnetic material and an external magnetic field. The magnetic microrobot is actuated by magnetic force or magnetic torque to generate motion under the action of an external magnetic field. At present, the magnetic field can be divided into a rotating field, gradient field, and oscillating field according to the form of actuating magnetically driven microrobot. The magnetic fields are usually generated by 3 types: permanent magnet, air core coil, and iron core coil. The permanent magnet can produce sizeable magnetic field intensity and magnetic field gradient. However, problems such as remanence and nonclosed magnetic fields must be addressed. Air core coils mainly include the Helmholtz coil and Maxwell coil [47], which can produce a uniform magnetic field and gradient magnetic field, respectively. Air core coils are convenient to model and control, but the magnetic field strength and working space are limited. The iron core coil consists of an iron core and copper wire, generating a gradient magnetic field to control the movement of microrobots directly [48]. Nevertheless, the iron core coil is unable to produce a uniform magnetic field, which is unsuitable for microrobot deformation and rotation. Usually, the magnetic-field-controlled microrobots fabricated by photopolymerization can be divided into rigid microrobots and soft microrobots.

#### Magnetically controlled rigid microrobots

**Fig. 3.**
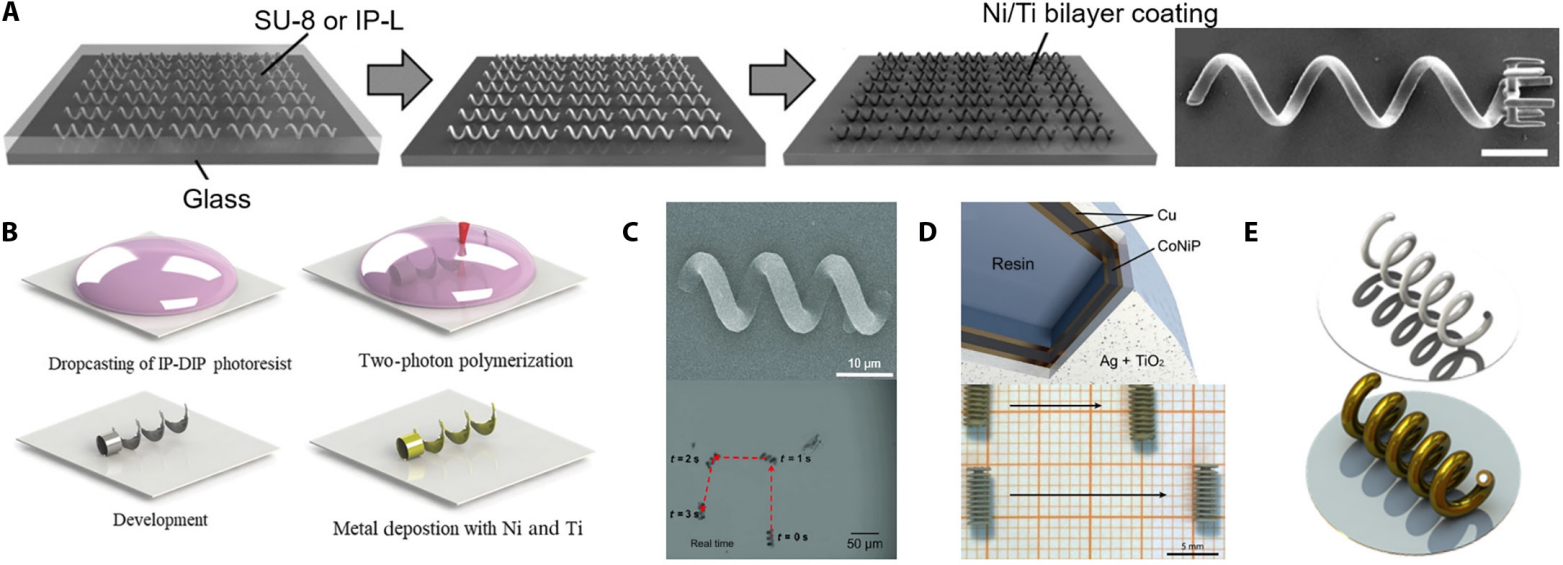
Magnetically controlled rigid microrobots. (A) Fabrication of the helical swimming micromachines and helical micromachine with a microholder [49]. Copyright 2012 John Wiley and Sons. (B) Fabrication of the capsule-type microrobot using 3D laser lithography [48]. Copyright 2018 John Wiley and Sons. (C) Scanning electron microscopy (SEM) image of microrobot and actuation trajectory [50]. Adapted from ref [50] with the permission under the terms of the CC license. (D) Microrobot structure and 2 models are driven in silicone oil [6]. Copyright 2019 Elsevier. (E) Wet metallized artificial bacterial flagella.

The rigid microrobots mainly refer to helical-type swimming microrobots. The helical-type swimming microrobots mainly consist of magnetic materials and helical bodies. The magnetic material obtains driving force through the external magnetic field, and the helical body simulates the bacterial flagella to carry out the spiral motion. The helical microstructures were obtained by photopolymerization, and the magnetic materials were deposited on the helical microstructures by electron beam evaporation. Tottori et al. [49] reported a helical-type microrobot fabrication method using 3D DLW and physical vapor deposition to fabricate helical-type microrobots with arbitrary shapes. It has a length of 8.8 μm and a diameter of 2.0 μm. As shown in Fig. 3A, DLW with negative-tone photoresist is employed to produce the helical-type microrobots. Nickel (Ni)/titanium (Ti) thin bilayer is deposited on the helical-type microrobot surface by electron beam evaporation. The helical-type microrobots use the body and microholder to transport cargo, and it has good drive controllability and biocompatibility. On this basis, Lee et al. [48] used a TPP technology to create 3D capsule-type microrobots. The microrobot is 65 μm in diameter and 290 μm in length. Fig. 3B shows that the capsule-type microrobot consists of the plunger and the cap. Ni and Ti layers are deposited on the plunger to respond to the magnetic field and enhance biocompatibility. The cap coats a Ti layer to avoid the effect of the magnetic field while controlling the plunger. The capsule-type microrobot exhibits a “pick-and-drop” motion with a broader range of application prospects. To improve the biocompatibility of microrobots. Giltinan et al. [50] proposed a 3D microprinting technique based on TPP that utilized iron platinum nanoparticles with ferromagnetic and biocompatible to produce 30-μm-long microrobots. Trimethylol propane-ethoxylated triacrylate polymer is printed directly through using TPP as shown in Fig. 3C. Instead of Ni/Ti thin bilayer, FePt nanoparticles are deposited by electron beam evaporation deposition, which enables magnetic field actuation and biological compatibility. Currently, many challenges lie ahead for untethered microrobots, such as poor biocompatibility and navigation in complex environments [51]. Helical-type microrobots are used not only in biomedical fields but also in the ecological environment field. Bernasconi et al. [6] combined SLA 3D printing and wet metallization to generate microrobots for water purification. Fig. 3D shows that different metallic layers are deposited on 3D printed parts using electroless and electrolytic deposition to impart desired functionalities. Taking advantage of the flexibility and versatility of electrolytic codeposition, pollutant photodegradation and bacteria killing are combined for the first time on the same device by covering a composite nanocoating containing titania nanoparticles in a silver matrix. From the perspective of water purification, the devices exhibit evident photocatalytic activity against water pollutants and antimicrobial activity against Gram-negative bacteria. On the basis of the above work, some scholars have optimized the fabricating method [52]. As shown in Fig. 3E, 3-turn helical structures with 5-μm pitch and 5-μm radius were fabricated. Only a layer of CoNiP alloy is deposited on the surface of the microrobot by electroless deposition, which enables the microrobot to have magnetically responsive properties. Moreover, the flexible and scalable fabricating approach also applies multiple metallic layers on the microrobot surface to enable 3D printed microstructures with multiple functions.

#### Magnetically controlled soft microrobots

**Fig. 4.**
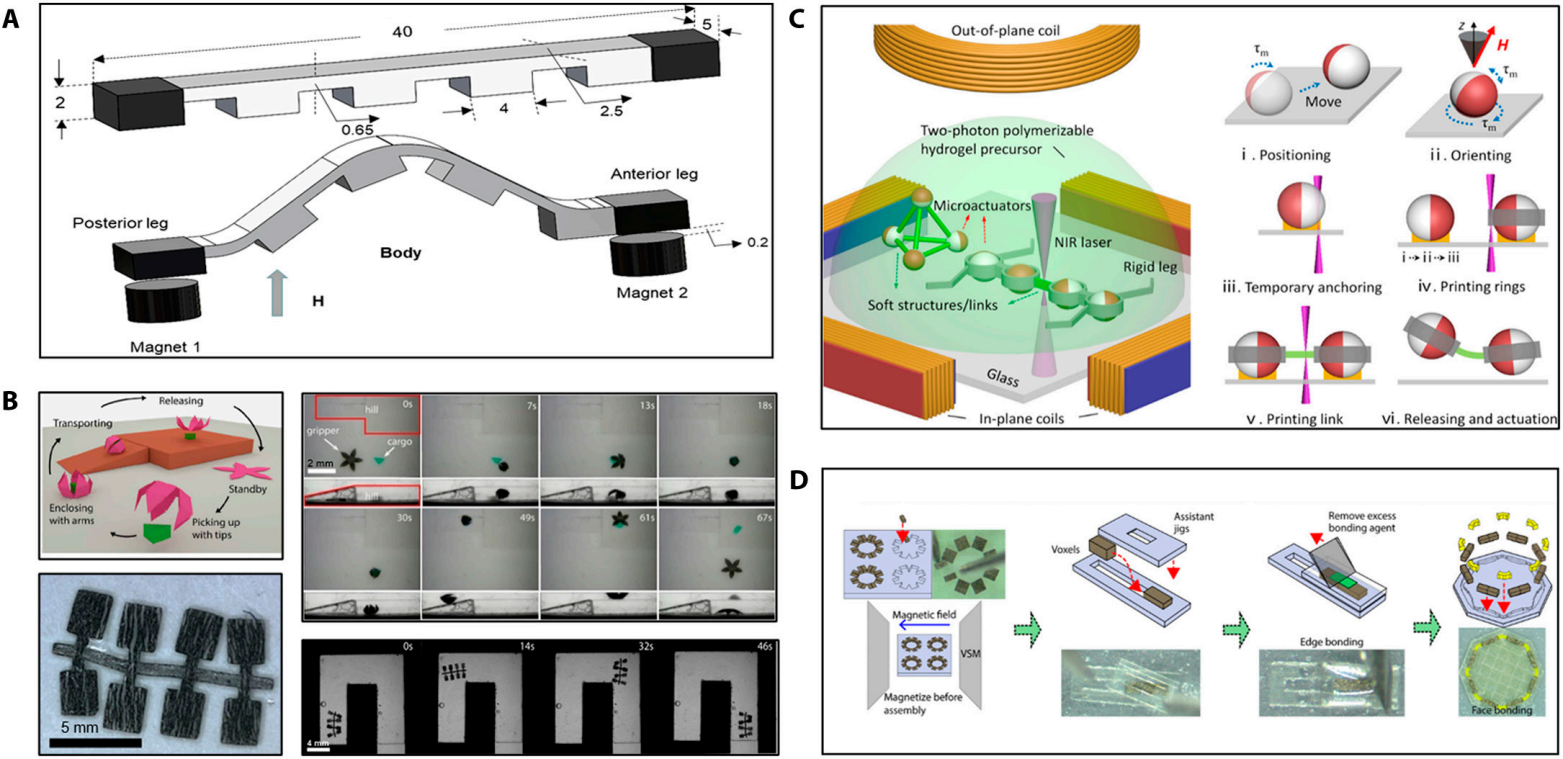
Magnetically controlled soft microrobots. (A) The model of untethered soft microrobot. (B) Schematic of microgripper; image of multilegged paddle-crawling microrobot; the movement image of microrobots [58]. Copyright 2019 the American Association for the Advancement of Science. (C) Schematic of the untethered multiarm magnetic microgripper fabrication [59]. Adapted from ref [59] with the permission under the terms of the CC-BY license. (D) Diagram of the multivoxel assembly process [60]. Copyright 2021 the American Association for the Advancement of Science. NIR, near-infrared; VSM, vibrating sample magnetometer.

Inspired by creatures in nature, soft microrobots are mainly biomimetic structure microrobots [53–55]. Soft microrobots are made of flexible materials and magnetic particle composite and hold great promise in many essential applications due to its inherent mechanical compliance that can enhance safety during operation [56]. Joyee et al. [57] printed a monolithic and untethered inchworm-inspired soft microrobot using a magnetic field-assisted projection SLA process. The soft robot is about 40 mm in length and 2 mm in height. As shown in Fig. 4A, the soft microrobot is directly printed by a 3D computer without any manual assembly or complex processing steps, and it is driven by the magnetic field. On the basis of ultraviolet (UV) lithography, Xu et al. [58] used controlled reorientation of magnetic particles to encode magnetic particles in planar materials with arbitrary 3D orientation, and the size of the printed microrobot is about 100 μm. The microrobot realizes multiple movements of multiarm grasping and multilegged crawling, as shown in Fig. 4B. In terms of the actuation method, the spatial magnetic field distribution output has improved with the improvement of the electromagnetic driving performance and optical microimaging technology. Although recent research has extensively promoted the fabricating of small-scale magnetic soft microrobots, integrating multiple material components remains a challenge. Hu et al. [59] employed TPP to selectively link Janus microparticles by 3D printing polymer microstructures and links. The microrobots are assembled and fabricated at the micrometer scale. As shown in Fig. 4C, each microactuator is positioned at the desired location by rolling and rotating to desired position and orientation by applying a magnetic field. Then, 3D printed links connect other temporarily fixed microactuators. Zhang et al. [60] produced a 3D magnetically driven soft microrobot based on multimaterial heterogeneous assembly. As shown in Fig. 4D, using TPP to fabricate models, various materials are cast into models to get multimaterial voxels. Last, the microrobot has multimaterial crystal and arbitrary 3D geometries by combining the voxels, which has a broad application prospect in the biomedical area.

Magnetic-field-controlled photopolymerization microrobots have many advantages, such as noninvasive, remote maneuverability, reconfigurability, and programmability of magnetic materials. However, the process of magnetization affects the rapid and continuous production of the microrobot. Moreover, the working spaces of the magnetic-field-controlled microrobots are also limited by the electromagnetic coil. Currently, microrobots with model-assisted voxel combinations of multiple materials have been designed, but the microrobots with multimaterials directly produced by photopolymerization still have many challenges. On the one hand, the light transmission properties of different materials and the complex operation when the materials are changed are the factors that restrict the development of photopolymerization to directly prepare multimaterial microrobots. On the other hand, photopolymerization multimaterial microrobots need to change light sources and materials, which leads to complicated operation.

### Optically controlled microrobots

Recently, optical-field-controlled microrobots have attracted more interest from researchers [61–63]. The optical field is expected to play an important role in controlling the cooperative motion of multiple microrobots due to the maturity and diversity of beam modulation and optical microscopy technologies [64]. According to different control principles, the optical-field-controlled microrobots are divided into optical tweezer (OT) control microrobots, optoelectronic tweezer (OET) control microrobots, and thermoresponsive material microrobots.

#### Microrobots controlled by OTs

**Fig. 5.**
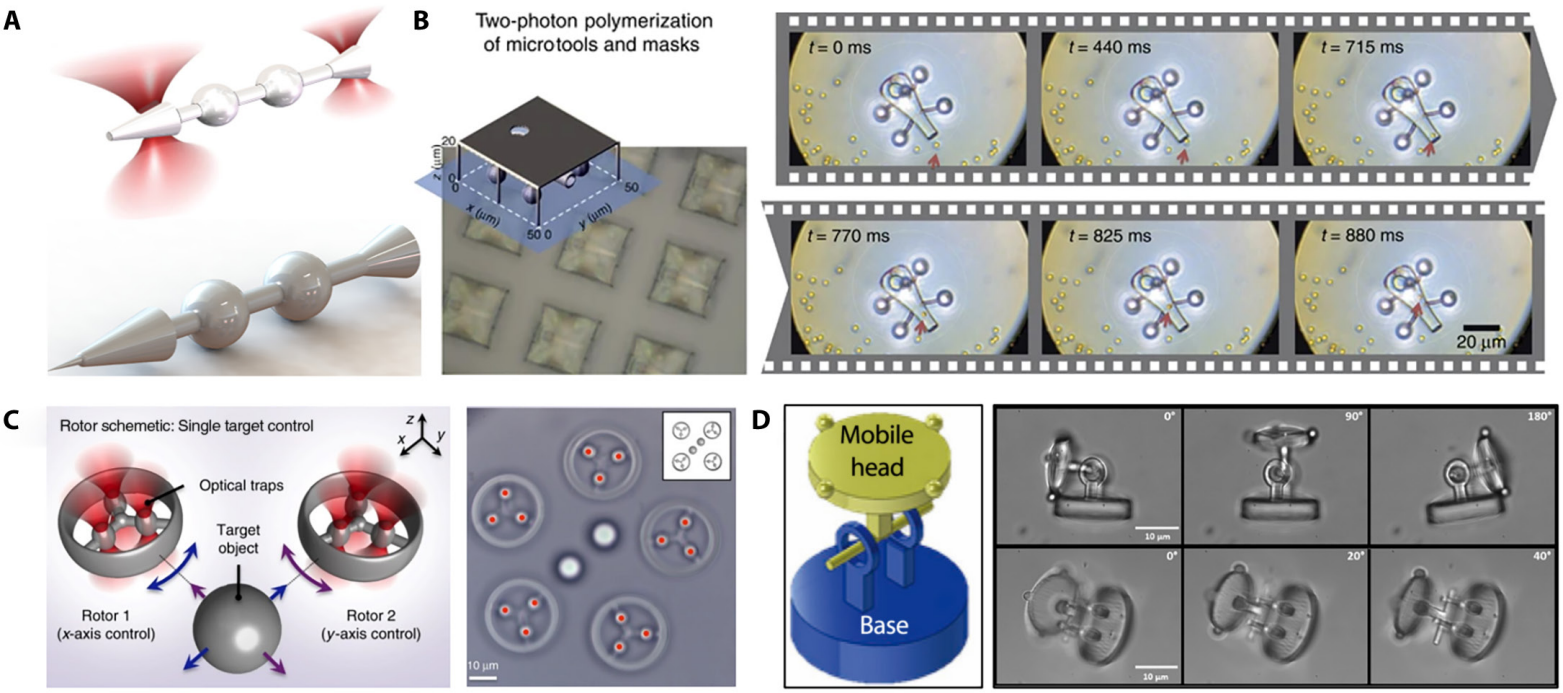
The OT-controlled microrobots. (A) Schematic of a tapered test particle and a probe tip [69]. (B) Schematic of microrobot fabrication; cargo transportation [70]. Adapted from ref [70] with the permission under the terms of the CC license. (C) Schematic of microrobots control; optical image of the experiment [71]. Adapted from ref [71] with the permission under the terms of the CC license. (D) The diagram of articulated microrobot; optical image of the articulated microrobot manipulation [72]. Copyright 2012 John Wiley and Sons.

OTs have been utilized to manipulate cells and biomolecules since those were invented in 1986 [65,66]. With the development of microrobots, the OT has been used to manipulate artificial microstructures [67,68]. OT relies on the momentum transfer of particles when light interacts with an object. The microrobot is controlled by optical gradient force and radiation pressure. In recent years, researchers have conducted several studies on optical polymerization microrobots controlled by the OT. Phillips et al. [69] employed TPP systems to fabricate and capture nonspherical particles. As shown in Fig. 5A, the optically trapped probe was fabricated from the shaped particles with linear tapers that can be used as a passive force clamp. The tip of passive force clamp is 3 μm in length and 200 nm in diameter. The passive force clamp is capable of applying a constant force over a displacement of several micrometers. As shown in Fig. 5B, Villangca et al. [70] designed a novel optical controlled microrobot using TPP. The microrobot is about 40 μm in size and has 2 openings with diameters of 6 and 8 μm. In addition to controlling microrobots by the OT, they are capable of loading and unloading cargo using photothermally induced convection currents within the microrobot body. Such type of microrobot is much faster than optical control. Optical-field-controlled microrobot can induce local microflow to control the target. For the first time, photopolymerization technology integrates multiple functions into a single stand-alone microrobot. Butaite et al. [71] designed a microrobot with a microrotor structure by utilizing TPP. Microrotors can be printed repeatedly with feature sizes as low as 100 nm by sweeping the beam across the photoresist. As shown in Fig. 5C, the OT is used to control the rotation of the microrotor to generate hydrodynamic force that can operate microtargets. Furthermore, photopolymerization technology shows clear advantages in the fabrication of microrobots with complex structures such as joints. Avci et al. [72] proposed that the joint microrobot based on TPP enables indirect manipulation of biological cells (Fig. 5D).

#### Microrobots controlled by OETs

**Fig. 6.**
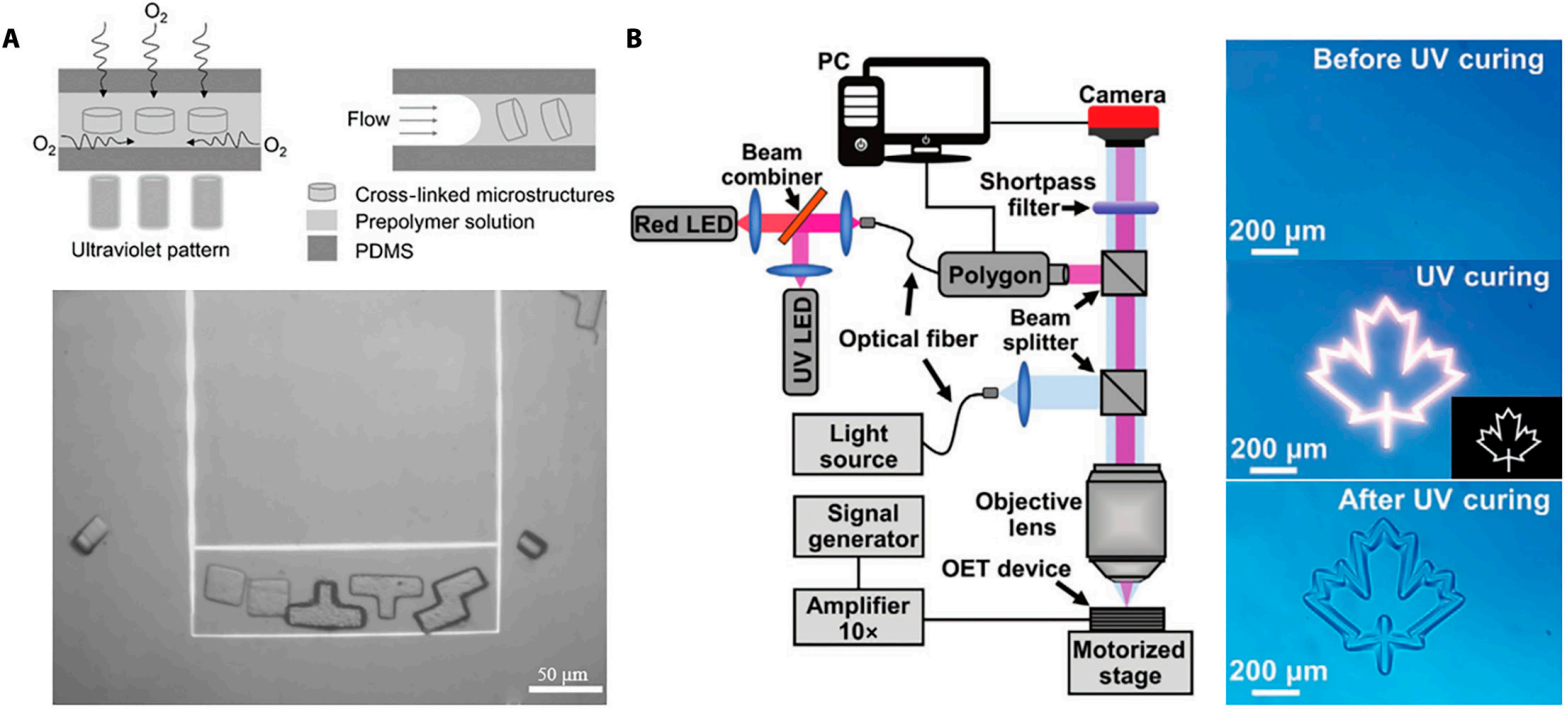
The OET controlled microrobots. (A) Schematic of microstructures fabricated in the polydimethylsiloxane (PDMS) device; the optically induced DEP manipulation and assembly [79]. Copyright 2016 John Wiley and Sons. (B) Diagram of the experimental setup; UV photopolymerization process of sol-form hydrogel solution [80]. LED, light-emitting diode. Copyright 2021 John Wiley and Sons.

OETs rely on optically induced-dielectrophoresis (DEP) force to actuate micro-objects [64]. The photoconductive substrate is used to induce a nonuniform electric field in the liquid medium above, and the electric field interacts with the sample in the medium to generate a DEP force that controls its motion [73,74]. Unlike the OT, the OET not only has a greater manipulation force that can control larger microrobots but also can operate multiple microrobots simultaneously [75–78]. Many scholars have recently combined the OET actuation with photopolymerization technology. Yang et al. [79] created complex and reconfigurable microrobots in hydrogels through DLP. As shown in Fig. 6A, using the OET, the building blocks with different units in the microfluidic chip can be arranged into desired geometric shapes. As shown in Fig. 6B, Zhang et al. [80] reported the topographical micropatterns based on OET in either “bottom-up” or “top-down” modes and then combined with in situ photopolymerizations to form permanent structures. Therefore, the OET has great promise in constructing artificial electronic and photonic microstructures.

#### Optically controlled thermoresponsive microrobots

**Fig. 7.**
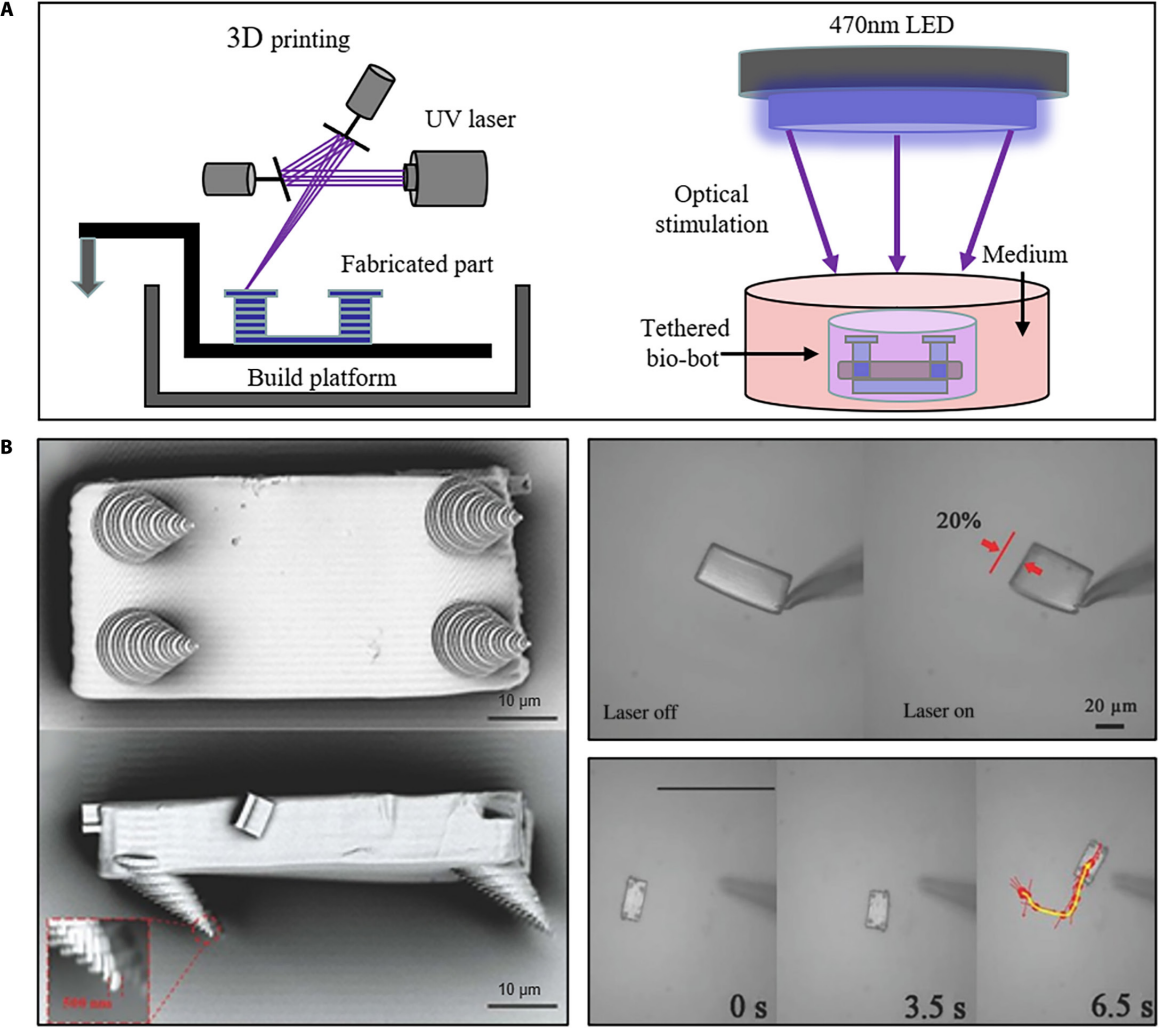
The optically controlled thermoresponsive microrobots. (A) Schematic of light-stimulated artificial muscle microrobot fabrication and stimulation. (B) SEM image of a microwalker; experiments of actuating microrobot [88]. Adapted from ref [88] with the permission under the terms of the CC license.

Apart from OT- and OET-controlled microrobots, microrobot fabricated by thermoresponsive material can also be controlled by optical fields [81]. The thermoresponsive materials, such as hydrogels [82–84] and liquid-crystal elastomers [85,86], can be mechanically deformed to drive microrobots. As shown in Fig. 7A, Raman et al. [87] utilized stereolithographic apparatus to assist in fabricating a light-stimulated artificial muscle microrobot. The microrobot obtained by this method can generate a passive tension force of 3.2 kPa and an active tension force of 0.56 kPa under external stimulation. Zeng et al. [88] proposed that a microrobot based on liquid crystalline elastomer (LCE) artificial muscles produced by TPP is entirely powered by an optical field. Fig. 7B illustrates a microrobot with 2 pairs of IP-Dip polymer legs and a minimum leg size of 500 nm. The LCE structure is heated above the glass transition temperature when illuminated with a focused green beam because the dyes in the LCE matrices absorb the green light. It shows approximately 20% contraction of the LCE body under illumination. With repeated on–off light signals, the structure exhibits a reversible contraction-swelling mode, which enables it to move along the glass substrate in the air.

As a rich renewable energy source, the optical field can realize the independent control of multiple microrobots. Moreover, optical fields show good performance in controlling microrobots in superficial locations such as close to the skin. However, high-energy optical fields can damage organisms and cause other diseases. The optical-field-controlled force is relatively small and not enough to drive microrobots of large size and weight. Complex and expensive control equipment also limits the development of optical-field-controlled microrobots.

### Acoustically controlled microrobots

Acoustics can be regarded as a robust and reliable source to manipulate microrobots remotely [89,90]. Compared with other fields, the acoustic field offers distinct relatively inexpensive in terms of strong penetration, high flexibility, and excellent biocompatibility [91,92]. Over the past few years, acoustic-field-controlled microrobots have gained extensive attention due to their broad application prospects [93–100]. Acoustic-field-controlled microrobots can be divided into 2 types according to the working principles: bubble propulsion and sharp-edge propulsion. This section aims to clarify the main features of the 2 types of microrobots.

#### Bubble propulsion

**Fig. 8.**
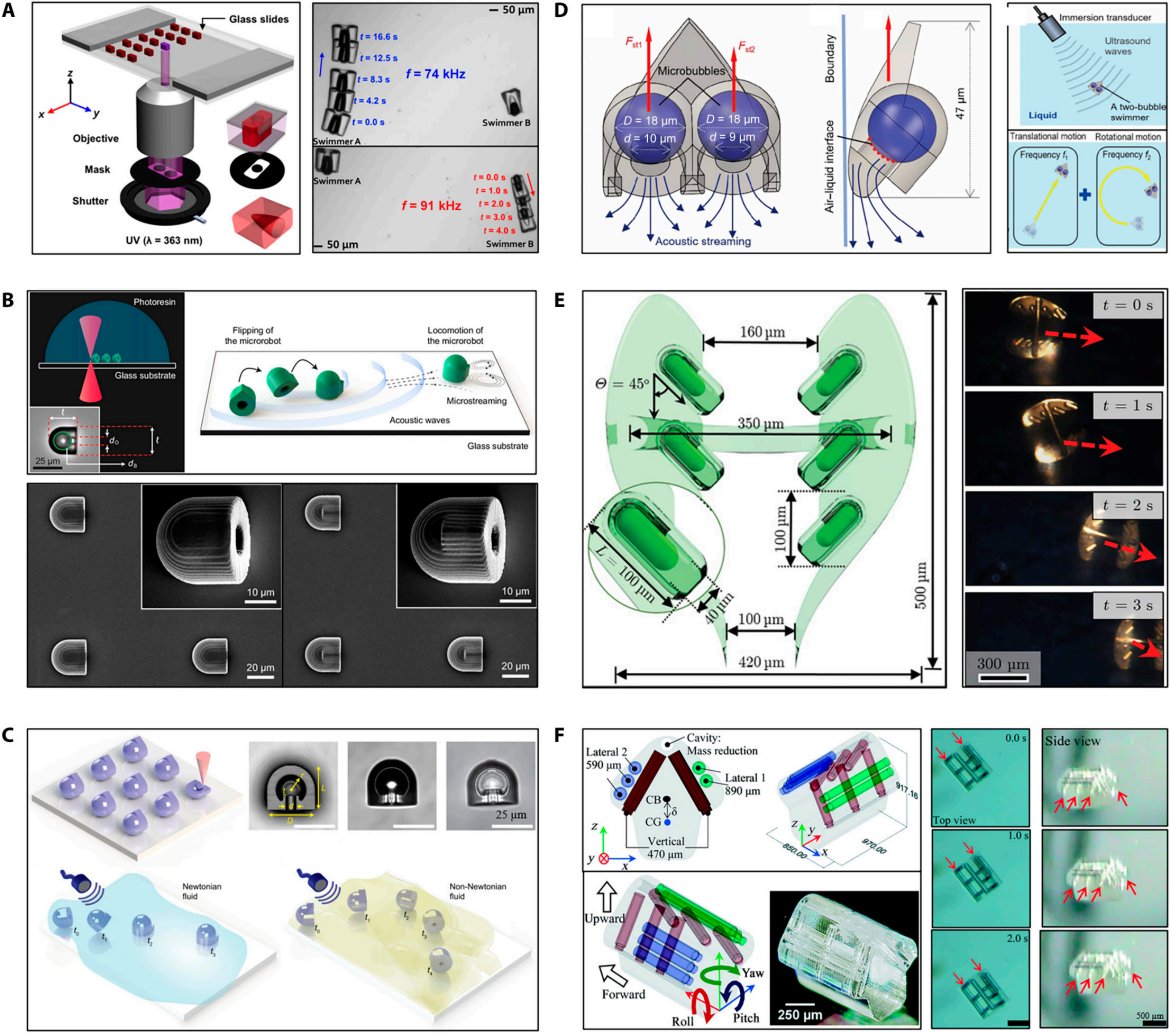
Acoustically controlled microrobots based on bubble propulsion. (A) Schematic of microswimmer fabrication; experiments of driving swimmers [97]. Adapted from ref [97] with the permission under the terms of the CC license. (B) Schematics of bullet-shaped microrobot fabrication and propulsion [101]. Adapted from ref [101] with the permission under the terms of the CC BY-NC-ND license. (C) Schematics of microrobot fabrication and propulsion; image of microrobot structure [102]. Adapted from ref [102] with the permission under the terms of the CC-BY license. (D) Design and control principles of a microswimmer [103]. Copyright 2021 Royal Society of Chemistry. (E) Schematic of the CeFlowBot microrobot; the ultrasound imaging of the microrobot motion [104]. Adapted from ref [104] with the permission under the terms of the CC license. (F) The diagram of microdrone; the experiments of actuating microrobot [105]. Copyright 2021 Royal Society of Chemistry. CB, center of buoyancy; CG, center of gravity.

The working principle of bubble propulsion is that the acoustic field excites the vibrations. The vibration is the most intense when the acoustic field frequency reaches the bubble resonance frequency. During bubble vibration, the liquid in the device is discharged and sucked as the bubble moves outward and inward. According to the nonlinear terms in the Navier–Stokes equation, the flow pattern generates a net momentum source that creates a driving force to push the entire device forward. Microrobots based on bubble propulsion is usually designed to have a streamlined structure with cavities. The cavity is used to generate and preserve bubbles, and the streamlined structure can improve the propulsion efficiency of the microrobots. Ahmed et al. [97] fabricated a microswimmer with an indentation structure using UV photopolymerization. The indentation of microswimmer is 50 to 100 μm in diameter as shown in Fig. 8A. The dents have different sizes of and determine the size of the air bubbles. When the microswimmer is submerged in the liquid-filled chamber, an air bubble can spontaneously become trapped in each of its indentations. By controlling the bubbles, individual microswimmer can be selective actuated. Aghakhani et al. [101] produced the bullet-shaped microrobot with a body length of 25 μm by TPP, and spherical voids are created in the microrobot to increase the stability of trapped bubbles. Fig. 8B shows that the microrobots complete a fast, unidirectional surface slipping locomotion on both flat and curved surfaces through the resonance of a bubble. Fig. 8C shows that the double reentrant microstructure cavity design is proposed. The structure not only increases the liquid repellency and the operational lifetime of the microrobots but also enhances the bubble stability [102].

Instead of a single bubble, Luo et al. [103] proposed a 2-bubble microswimmer via TPP. The overall shape of the microswimmer is triangular, and the back is composed of 2 bubbles with a diameter of 18 μm. As shown in Fig. 8D, the microswimmer can be propelled and steered using a single ultrasound transducer since the 2 bubbles have different diameters [103]. The control of the microrobot depends on the bubble vibration, and the presence or absence of the bubble is related to the actuation time. To improve the actuation time of microrobots, Mohanty et al. [104] fabricated the CeFlowBot microrobot with a complex structure of 6 bubble arrays using TPP, as shown in Fig. 8E. The propulsion speeds and actuation time of the microrobots have been increased because of multiple bubbles being actuated in tandem. Liu and Cho [105] have also fabricated microrobots with complex structures through TPP. Fig. 8F shows that the 3 microtubules are embedded in the microrobot body, thereby obtaining 3 bubbles and realizing the 3D movement of the microrobot by selectively exciting the bubble vibration. However, in low Reynolds, the motion behavior of bubble propulsion microrobots is complex. When the microbubble size is less than 30 μm, the microbubble vibrates near the solid wall due to nonlinear acoustic forces [106]. These kinds of acoustic-field-controlled microrobots have not been thoroughly studied, and directional control is still challenging.

#### Sharp-edge propulsion

**Fig. 9.**
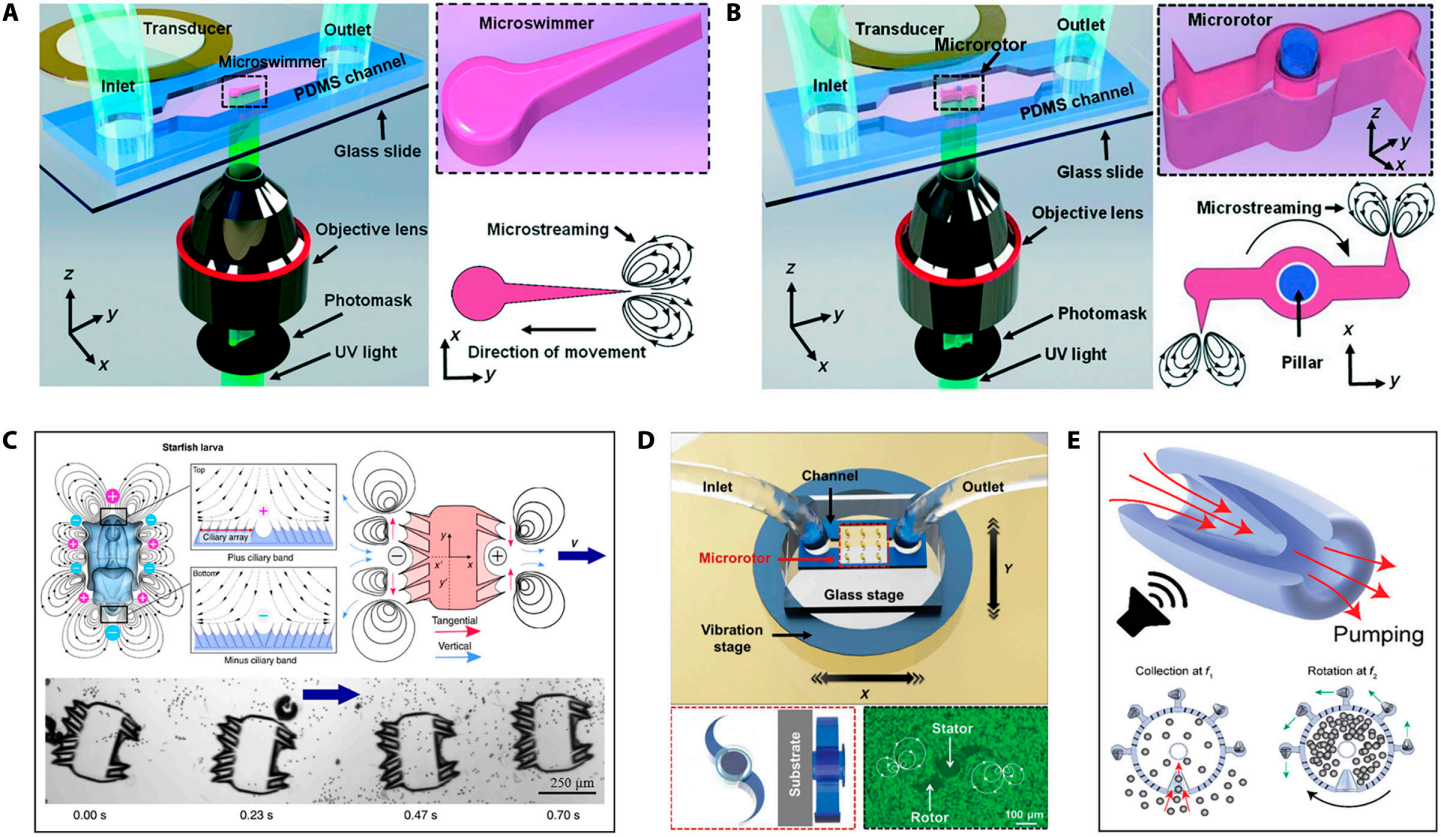
Acoustically controlled microrobots based on sharp-edge propulsion. (A) Schematic of microswimmer fabrication and actuation [110]. Copyright 2017 Royal Society of Chemistry. (B) Schematic of microrotor fabrication and actuation [111]. Copyright 2016 Royal Society of Chemistry. (C) Schematic of artificial microrobot; the image of driving microrobot [112]. Adapted from ref [112] with the permission under the terms of the CC license. (D) Schematic of microfluidic multiple microrotor setup; The image of microstreaming flow lines [114]. Copyright 2020 John Wiley and Sons. (E) Schematic of the microrobot actuation [115]. Adapted from ref [115] with the permission under the terms of the CC license.

In nature, many microorganisms have flagellar structures and are powered by flagellar oscillations [107]. The flagellar structure inspires sharp-edge propulsion [108,109]. Microrobots based on sharp-edge propulsion are usually made with sharp structure, and the sharp structure can generate reverse rotating eddy currents around the tip of the acoustic field to control the motion of the microrobots. Some sharp edges also wobble in the acoustic field, providing power for the microrobots. The sharp-edge propulsion microrobots have been fabricated by photopolymerization. Kaynak et al. [110] produced a microrobot with flagellar structures in the microchannel by UV photopolymerization, as shown in Fig. 9A. The exposure time of a microrobot only needs approximately 50 ms. Microrobot is about 180 μm in length, 60 μm in width (at the head of the swimmer), and 45 μm in height. Subsequently, Kaynak et al. [111] fabricated a polymeric microrotor with predefined oscillating sharp-edge structures in situ by applying a patterned UV light that polymerizes a photocrosslinkable polyethylene glycol solution. As shown in Fig. 9B, single-step in situ fabrication of microrotors with complex structures is employed by UV photopolymerization in a microchannel. Piezoelectric transducer enables acoustofluidic actuation of the microrotor through microflows induced by the oscillations of sharp-edge structures. Inspired by ciliary bands on the surface of the starfish larvae, Dillinger et al. [112] fabricated arrangements of cilia using UV photopolymerization. Fig. 9C shows that the microrobot has 2 to 8 cilia on each side, and the ciliary bands can be obtained by one-step fabricating. The movement of the microrobot is realized by acoustically controlled small-amplitude oscillations of the cilia.

Similarly, TPP has exhibited tremendous advantages in realizing exquisite microstructure design [113]. As shown in Fig. 9D, Zhou et al. [114] fabricated multiple microrotors adopting TPP. The microrotor consists of a stator and a rotor: The diameter of the stator was 100 μm, and the distance between 2 rotors was 360 μm. An axially movable rotor is more easily obtained in this way. The tunable acoustic vibration of the multiple microrotors is achieved using a piezoelectric vibration stage, and the rotational speed of microrotors with sharp curved tips can reach 1,600 rpm in water. This approach enables not only easy microrotor fabrication but also simple remote actuation of multiple rotors, which provides promising prospects in various microfluidic manipulation applications. To fabricate microrobots with more complex structures, Kaynak et al. [115] developed a microjet engine microrobot using TPP. Fig. 9E shows that the microrobot consists of a cylindrical shell wrapped in a flexible conical wedge made of hydrogel and polyethylene glycol diacrylate. It performs micromanipulation through local body deformation that produces fluid, such as on-demand collection, encapsulation, and processing of microscopic samples. This paves the way for the development of soft microrobots with tailored performance and functionality.

The acoustic field can realize the remote driving of microrobots and is a reliable actuation source. Furthermore, the noninvasiveness to the living body makes the acoustic-field-controlled have broad application prospects. However, it is difficult for the acoustic field to achieve accurate movement and control of the microrobots alone, so it is necessary to combine other control methods to achieve the desired control precision. Currently, acoustic-field-controlled is still in its early stages. In the acoustic field environment, the forces on the microrobots and the targets are complex, and the utilization of the acoustic field to achieve precise control of the microrobots requires continued research. In addition, we should take the practicability of acoustic-field-controlled microrobots instead of focusing on the effective control microrobots. Realizing the real application of acoustic-field-controlled microrobots *in vivo* environment may be the main goal of the researchers.

### Electrically controlled microrobots

Electric field power is one of our most common power sources. Electric fields can not only be applied to large-scale types of equipment but also have great potential in controlling microscale microrobots [116]. In the electric field, the control of particles by the electrophoretic motion of charged particles or molecules, such as electrophoresis and electroosmosis of diodes [117–120]. Bipolar electrochemical phenomena in electric fields can also be adapted to control particles [121].

**Fig. 10.**
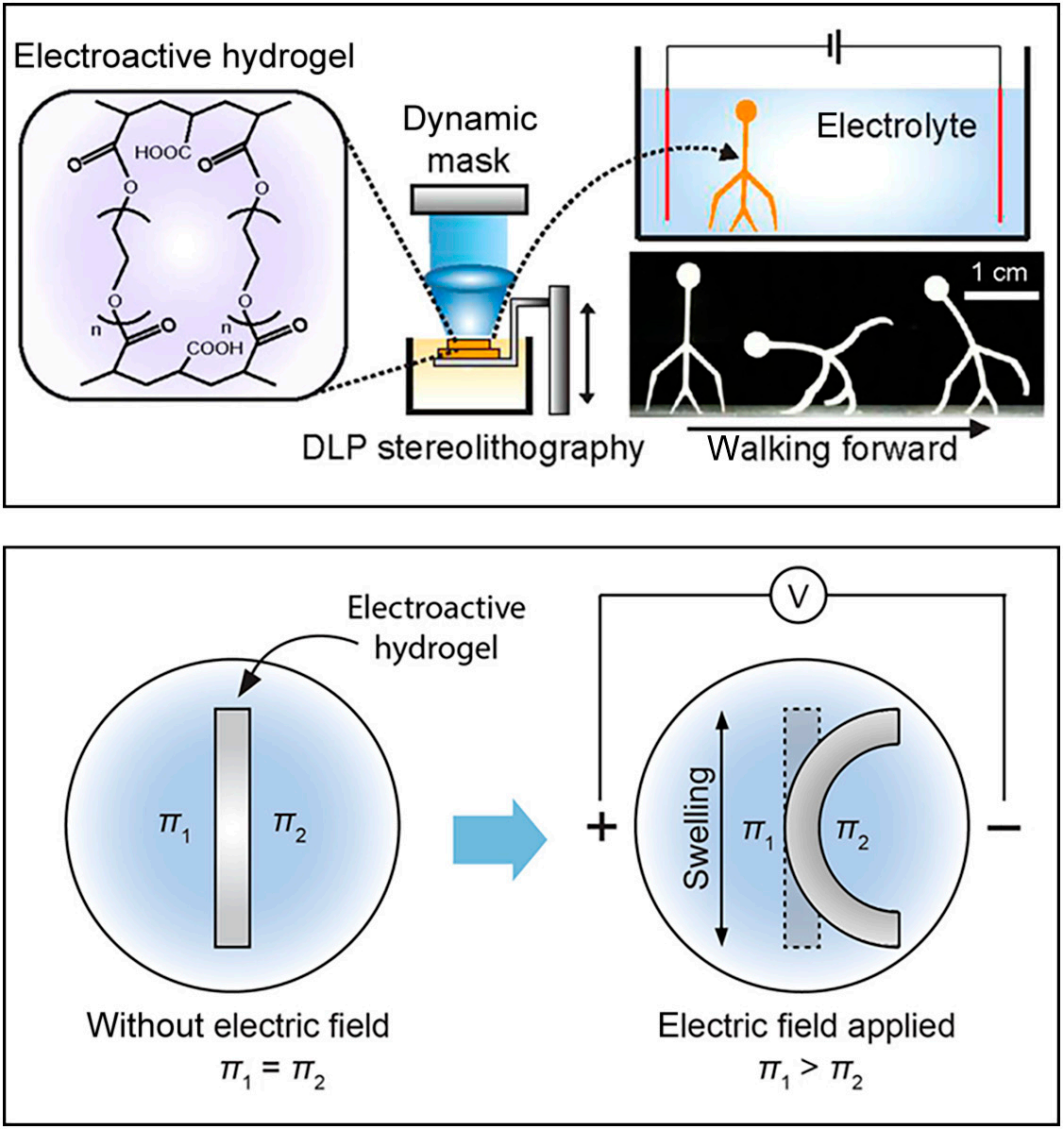
Schematic and the bending mechanism of the electroactive hydrogel microrobot [123]. Copyright 2018 American Chemical Society.

Moreover, the soft microrobot made of electroactive smart materials that can respond to electrical stimulation has attracted much attention [122]. Han et al. [123] designed soft microrobots with electroactive hydrogels using DLP. The thickness of the “body”, “legs”, and “arms” of microrobot can reach 220, 300, and 220 μm, respectively. As shown in Fig. 10, electroactive hydrogel microrobots are placed in an electrolyte solution (phosphate-buffered saline) to generate ionization, and the microrobot becomes a network anionic. The anionic network forms osmotic pressure with surrounding cations, and the osmotic pressure reaches equilibrium [124–127]. The balance is broken when the microrobot is placed in the electric field and the shape of the microrobot changes. The microrobots can achieve bidirectional motion by changing the direction of the electric field. However, a drawback of electric actuation is the presence of electrodes in the work area, which may limit the bio-oriented applications. At present, electroactive hydrogel microrobots have been obtained through DLW [124], template-assisted UV curing [128], and laser cutting [129]. Nevertheless, there are few electroactive hydrogel microrobots designed by photopolymerization, and it is believed that photopolymerization will be widely used for microrobot applications in the future.

Electric field control is a contact-free and fuel-free driving system, which can control the microrobot at the microscopic scale using dielectric electrophoresis force. However, the electric field is harmful to biological targets. The rapid attenuation of the electric field with distance leads to the shorter working distance of microrobots. Moreover, the electric field may not be compatible with the body fluids containing high ionic medium, such as tissue fluid or blood, causing harm to the organism.

## Conclusion and Outlook

In this review, we focus on state-of-the-art works on fabricating the field-controlled microrobots by photopolymerization, including the different photopolymerization approaches, the materials, and the different actuating field forces. Photopolymerization has the advantages of fast-printing velocity, high accuracy, and high surface quality, which make it one of the most commonly utilized field-controlled fabrication technologies. In contrast, the template-assisted electrodeposition method and the physical vapor deposition method are highly limited in printing speed and precision. Under different actuating fields, photopolymerization microrobots can accomplish complex and various tasks, which allows their massive applications in the biomedical field.

There are 2 main future developing directions of the field-controlled microrobots fabricated by photopolymerization, including the smart materials and capabilities for real applications. In terms of printing materials, smart materials with self-actuating, self-sensing, and shape-changing abilities in response to external stimuli offer more possibilities for photopolymerization technology. At the same time, the development of fabrication technology has promoted the fusion of different intelligent materials, enabling microrobots to combine multiple capabilities to achieve various behaviors. Moreover, the research on microrobots is still executed in the laboratory but away from the application. Both fabrication and performance aspects should be considered and optimized to apply microrobots from the laboratory to *in vivo* clinical. The performance of photopolymerization technology is one of the essential precautions that should be enhanced, such as the development of field-assisted photopolymerization technology to improve multimaterial fabricating capabilities and the producibility of complex designs. Besides, the biocompatibility and biodegradability of microrobots are vital to be considered to minimize the impact and damage of microrobots on the human body. It is also critical to select suitable and effective control methods to meet the requirements of complex environments and tasks *in vivo* clinical. Collaborative control of microrobots through multiple combinations of physics may be a viable approach.

It is convincing that with the improvement of printing technology, the development of new materials, and the rational design of multiple control methods, field-controlled microrobots fabricated by photopolymerization with better performance will usher in vigorous development. As more and more field-controlled microrobots continue to be designed and fabricated by photopolymerization, we expect that more progress for microrobots applied in fields of biomedicine, environmental governance, aerospace, and other fields will be made soon.

## Data Availability

The data used to support the findings of this study are available from the corresponding author upon request.
